# Stem cell-based therapies for ischemic stroke: a systematic review and meta-analysis of clinical trials

**DOI:** 10.1186/s13287-020-01762-z

**Published:** 2020-06-26

**Authors:** Zhonghao Li, Xiaoke Dong, Min Tian, Chongchong Liu, Kaiyue Wang, Lili Li, Zunjing Liu, Jinmin Liu

**Affiliations:** 1 Department of Neurology, Dongfang Hospital Beijing University of Chinese Medicine, No. 6 Fangxingyuan 1st Block, Fengtai District, Beijing, 100078 China; 2 Department of Neurology, China-Japan Friendship Hospital, Ying Hua Dong Jie, Beijing, 100029 China

**Keywords:** Cell transplantation, Cellular delivery, Cerebral infarction, Stroke, Neurological deficit, Activities of daily living

## Abstract

Recently, extensive researches about stem cell-based therapies for ischemic stroke have been published; our review evaluated the efficacy and safety of stem cell-based therapies for ischemic stroke. Our review was registered on PROSPERO (http://www.crd.york.ac.uk/PROSPERO), registration number CRD42019135805. Two independent observers searched PubMed, EMBASE, Cochrane Library (Cochrane Database of Systematic Reviews, Cochrane Central Register of Controlled Trials), and Web of Science (Science Citation Index Expanded) for relevant studies up to 31 May 2019. We included clinical trials which compared efficacy outcomes (measured by National Institutes of Health Stroke Scale (NIHSS), modified Rankin scale (mRS), or Barthel index (BI)) and safety outcomes (such as death and adverse effects) between the stem cell-based therapies and control in ischemic stroke. We performed random effect meta-analysis using Review Manager 5.3. Our review included nine randomized controlled trials (RCTs) and seven non-randomized studies (NRSs), involving 740 participants. Stem cell-based therapies were associated with better outcomes measured by NIHSS (mean difference (MD) − 1.63, 95% confidence intervals (CI) − 2.73 to − 0.53, *I*^*2*^ =60%) and BI (MD 14.68, 95% CI 1.12 to 28.24, *I*^*2*^ = 68%) in RCTs, and by BI (MD 6.40, 95% CI 3.14 to 9.65, *I*^*2*^ = 0%) in NRSs. However, the risk of bias was high and the efficacy outcomes of RCTs were high heterogeneity. There was no significant difference in mortality between the stem cell group and the control group. Fever, headache, and recurrent stroke were the most frequently reported adverse effects. Our review shows that stem cell-based therapies can improve the neurological deficits and activities of daily living in patients with ischemic stroke.

## Introduction

Stroke is the second most common cause of death and disability in the world, leading to a heavy burden on patients, family, and society [1]. As a predominant stroke subtype, ischemic stroke constituted 69.6% among all subtypes of incident stroke according to the national epidemiological survey of stroke in China [2]. At present, intravenous recombinant tissue plasminogen activator and endovascular mechanical thrombectomy are effective at the hyperacute phase, but they are hampered by the narrow time window and strict indications [3, 4]. Patients who fail to receive these managements may be left with a residual deficit. Although rehabilitation can aid functional recovery and brain reorganization, the effects are still limited [5]. Pharmacological attempts to stimulate repair and neuroprotection have been widely investigated in experimental studies but few have been effective in clinical use [6]. More therapeutic approaches are needed.

Infarction causes an acute loss of different cells such as neurons and glial cells in the brain. The initial stem cell-based therapies were aimed toward a cell replacement strategy and have been demonstrated in laboratory [7]. However, a number of studies showed that the beneficial effects are mediated by indirect mechanisms, such as attenuating inflammation, reducing scar thickness, enhancing autophagy, normalizing microenvironmental/metabolic profiles, releasing trophic factors and cytokines, and possibly replacing damaged cells [8–10]. In 2005, Bang and colleagues firstly transplanted autologous mesenchymal stem cell to five stroke patients and patients’ functional recovery was improved in 1 year follow-up with no cell-related adverse effect [11]. Since then, a number of clinical trials have been conducted to verify the efficacy and safety of stem cell-based therapies for ischemic stroke with different stem cell types, doses, and delivery routes at different phases of stroke [12]. But the outcomes in different stroke scales are inconsistent [13, 14]. In this study, we attempted to investigate the effectiveness and safety of stem cell-based therapies for ischemic stroke.

## Methods

Our review was registered on PROSPERO, the international prospective register of systematic reviews (http://www.crd.york.ac.uk/PROSPERO), registration number CRD42019135805. The PRISMA checklist is available in Additional file 1.

### Inclusion criteria and exclusion criteria

The inclusion criteria for the studies were (1) patients with ischemic stroke confirmed by computerized tomography or magnetic resonance imaging scan regardless of the disease phase; (2) interventions involved stem cell-based therapies, regardless of stem cell types and the delivery routes; (3) comparison involved standard treatment for the management of stroke, injection of placebo or no treatment; and (4) efficacy outcomes (measured by National Institutes of Health Stroke Scale (NIHSS), modified Rankin scale (mRS), or Barthel index (BI)) and safety outcomes (including death and other adverse effects) were reported. Exclusion criteria were (1) patients aged over 80 years, (2) single-arm studies, or (3) outcome data could not be extracted.

### Search strategy

Two independent observers (M.T. and X.D.) searched the following electronic bibliographic databases: PubMed, EMBASE, The Cochrane Library (Cochrane Database of Systematic Reviews, Cochrane Central Register of Controlled Trials), and Web of Science (Science Citation Index Expanded) from inception to May 31, 2019. The keywords used in the search strategy included “stem cells,” “cell therapy,” “stroke,” “cell transplantation,” and “brain infarction.” The search strategy for PubMed is available in Additional file 2. The search terms were adapted for use with other databases in combination with database-specific filters for clinical trials, where these were available. There was no language restriction. The search was re-run on Jan 20, 2020, just before the final analysis, and further studies were retrieved for inclusion, and there was no additional included study.

### Selection of studies

Titles and/or abstracts of all relevant studies were screened independently by two reviewers (K.W. and L.L.) to identify studies that met the above inclusion criteria. The full text of these potentially eligible studies was retrieved and independently assessed for eligibility by two review team members. Any disagreement between the two reviewers regarding the eligibility of a study was resolved through discussion with a third reviewer (C.L.).

### Data extraction

A standardized, pre-piloted form was used to extract data from the included studies for assessment of study quality and evidence synthesis. Extracted information included country in which the study was conducted, study population and participant demographics, details of the intervention and control conditions, outcomes and times of measurement, and information for the assessment of the risk of bias. Two reviewers (X.D. and M.T.) extracted data independently; discrepancies were identified and resolved through discussion (with a third reviewer (Zh.L.) when necessary). Missing data were requested from study authors.

### Quality of assessment

Assessment of the quality of the included studies was performed using the method recommended by Cochrane Handbook for Systematic Reviews of Interventions [15]. For randomized controlled trial (RCT), the Cochrane risk of bias tool was used. This method comprised assessments of the risk (low risk, high risk, or unclear risk) of potential bias in seven domains: random sequence generation, allocation concealment, blinding of outcome assessment, blinding of participants and personnel, incomplete outcome data, selective reporting, and other biases, such as the baseline, source of funding, and academic biases. For non-randomized study (NRS), the Newcastle-Ottawa Scale was used [16]. This method comprised assessments of the risk of potential bias in three domains: selection, comparability, and outcome. Study ratings of seven to nine stars corresponded to high quality, five to six stars to moderate quality, and four stars or less to low quality. Two reviewers (C.L. and X.D.) independently assessed the quality of the included trials. Disagreements between the reviewers over the risk of bias were resolved by discussion with a third reviewer (Zh.L.).

### Statistical analysis

We provided summaries of intervention effects for each study by calculating risk ratios (for dichotomous outcomes) or mean differences (for continuous outcomes). For studies that used the same type of intervention and comparator, with the same outcome measure, we pooled the results using a random effect meta-analysis, with mean differences (MD) for continuous outcomes and risk ratios (RR) for binary outcomes, and calculated 95% confidence intervals (CI) and two-sided *P* value for each outcome. Studies in different types (RCT or NRS) were pooled separately. Heterogeneity between the studies was assessed using both of the chi-square test and the *I*^*2*^ statistic, and in the *I*^*2*^ value, more than 50% were considered to represent substantial heterogeneity. We conducted sensitivity analyses based on study quality. We used stratified meta-analyses to explore heterogeneity in effect estimates according to study quality, study populations, the logistics of intervention provision, and intervention content. Review Manager 5.3 software was used for statistical analysis.

## Results

### Results of the search

**Fig. 1:**
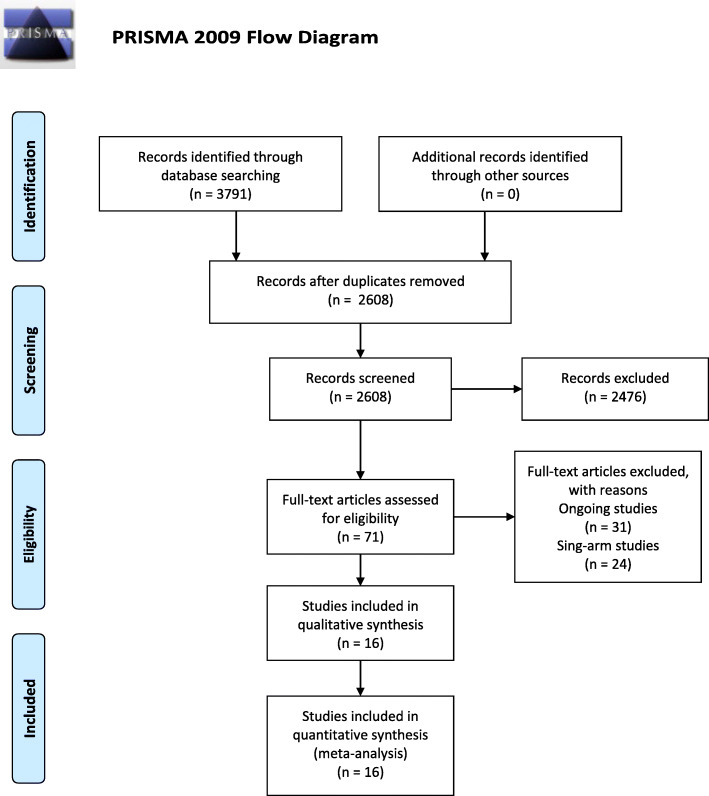
Flow diagram of this meta-analysis according to PRISMA 2009

A total of 3791 records were identified and no additional record. One thousand One hundred eighty-three records were excluded as duplicates. An additional 2476 references were excluded because they were not relevant. After full-text review of the remaining 132 references, referring to 71 studies, we excluded 31 ongoing studies and 24 single-arm studies (Additional file 2). Finally, we included 16 studies [11, 17–31] involving 740 participants in this review (Fig. 1).

### Characteristics of included studies

Of the sixteen studies, nine were RCTs and seven were NRSs, fifteen were written in English and one was written in Chinese. Most of the studies were in Asia: six in India, three in China, and two in South Korea. Most of the studies included patients with NIHSS more than 4, involving the middle cerebral artery territory, and with at least 8 weeks of follow-up. All studies used adult human non-neural stem cells: five bone marrow-derived mesenchymal cells (MSCs), six bone marrow-derived mononuclear cells (BMMNCs), one peripheral blood stem cells (PBSCs), one multipotent adult progenitor cells (MultiStem), one aldehyde dehydrogenase cells (ALD-401), one both endothelial progenitor cells (EPCs) and MSCs, and one both MSCs and BMMNCs. The most used administration route was intravenous injection. All studies reported efficacy outcomes and safety outcomes. The characteristics of the included studies are shown in Table 1.

**Table 1:** Characteristics of included studies

Author year	Study type	Country	Cases (SC/control)	Eligibility criteria (mean NIHSS)	Time onset to SC infusion	Cell type	Cells dose	Route of administration	Other treatments			Follow-up	Outcomes
									Reperfusion interventions (%)	Antithrombotic treatment (%)	Rehabilitation theory (%)		
Bang 2005 [11]	RCT	South Korea	5/25	NIHSS ≥ 7 (10.6), MCA territory	32 to 61 days	Autologous MSCs	5 × 10^7^, twice	i.v.	Thrombolytics (20%)	Antiplatelet (76.7%), anticoagulant (23.3%)	Mean 57.3 days (100%)	12 months	Infarcted volumes, NIHSS, mRS, BI, safety outcomes
Bhatia 2018 [17]	RCT	India	10/10	NIHSS > 7 (10.5), MCA territory	Mean 10 days	Autologous BMMNCs	Mean 6.1 × 10^8^	i.a. ipsilateral MCA	Thrombolytics (5%)	NG	NG	6 months	NIHSS, mRS, BI, safety outcomes
Chen 2014 [18]	RCT	China	15/15	NIHSS 9–20 (9.3) with motor deficits, MCA territory	6 months to 5 years	Autologous PBSCs	3–8 × 10^6^	Stereotaxic implantation	NG	Antiplatelet (100%)	Mean 75.6 days (100%)	12 months	NIHSS, mRS, ESS, EMS, safety outcomes
Fang 2019 [19]	RCT	China	10/6	NIHSS ≥ 7 (14.9), MCA territory	Mean 33.5 days	Autologous EPSs (50%), autologous MSCs (50%)	2.5 × 10^6^/kg, twice	i.v.	None	Antiplatelet (62.5%), anticoagulant (37.5%)	NG	4 years	NIHSS, mRS, BI, SSS, safety outcomes
Hess 2017 [20]	RCT	UK and USA	67/62	NIHSS 8–20 (12.8), MCA territory, infarct size between 5 and 100 cc^3^	24 to 48 h	Allogeneic multipotent adult progenitor cells (MultiStem)	1.2 × 10^9^	i.v.	Thrombolytics (45.7%), thrombectomy (22.5%), both (13.2%)	NG	NG	12 months	NIHSS, mRS, BI, safety outcomes
Jin 2017 [21]	RCT	China	10/10	NIHSS 4–31 (10.7)	3 weeks to 5 months	Autologous BMMNCs	1 × 10^7^	Subarachnoid infusion	NG	Antiplatelet	NG	7 years	BI, mRS, FIM, Fugl-Meyer motor scale, adverse reactions
Lee 2010 [22]	RCT	South Korea	16/36	Modified NIHSS ≥ 7 (10.63), MCA territory	4 to 9 weeks	Autologous MSCs	5 × 10^7^, twice	i.v.	Thrombolytics (34.5%)	NG	More than 2 weeks (38.4%)	5 years	mRS, safety outcomes
Prasad 2014 [23]	RCT	India	60/60	NIHSS ≥ 7 (11), anterior circulation	Mean 18.5 days	Autologous MSCs	Mean 2.8 × 10^8^	i.v.	None	NG	NG	12 months	NIHSS, mRS, BI, safety outcomes
Savitz 2019 [24]	RCT	USA	29/19	NIHSS ≤ 22 (10), mRS ≥ 3, MCA territory	13 to 19 days	Autologous bone marrow derived ALDHbr cells (ALD-401)	Mean 3.08 × 10^6^	i.a. ipsilateral ICA	Thrombolytics (27.6%), thrombectomy (17.2%)	NG	NG	12 months	mRS, NIHSS, BI, EQ-5D, safety outcomes
Bhasin 2012 [25]	NRS	India	12/12	NIHSS 4–15	3 months to 2 years	Autologous BMMNCs	Mean 5.46 × 10^7^	i.v.	NG	NG	8 weeks (100%)	24 weeks	Modified BI, Fugl-Meyer scale for upper limbs, MRC grade, Ashworth tone scale, safety outcomes, functional MRI
Bhasin 2013 [26]	NRS	India	20/20	NIHSS 4–15	3 months to 2 years	Autologous MSCs (30%), autologous BMMNCs (70%)	Mean 5.54 × 10^7^	i.v.	NG	NG	8 weeks (100%)	24 weeks	Modified BI, Fugl-Meyer scale for upper limbs, MRC grade, Ashworth tone scale, Edinburgh handedness inventory, safety outcomes
Bhasin 2016 [27]	NRS	India	10/10	NIHSS 4–15	3 months to 1.5 years	Autologous BMMNCs	Mean 6.28 × 10^7^	i.v.	NG	NG	8 weeks (100%)	8 weeks	Modified BI, Fugl-Meyer scale for upper limbs, MRC grade, Ashworth tone scale, Edinburgh handedness inventory, serum growth factors, safety outcomes
Bhasin 2017 [28]	NRS	India	6/6	NIHSS 4–15	3 months to 2 years	Autologous MSCs	Mean 5–6 × 10^7^	i.v.	NG	NG	8 weeks (100%)	4 years	Modified BI, Fugl-Meyer scale for upper limbs, MRC grade, Ashworth tone scale, safety outcomes
Ghali 2016 [29]	NRS	Egypt	21/18	NIHSS 4–20 (10.8), MCA territory	12 to 32 days	Autologous BMMNCs	1 × 10^6^	i.a. ipsilateral ICA	None	NG	NG	12 months	NIHSS, mRS, BI, modified and standardized Arabic version of the Comprehensive Aphasia Test, radiological and safety outcomes
Meng 2009 [30]	NRS	China	60/60	Ischemia stroke	Mean 21 days	Autologous MSCs	Mean 2.97 × 10^9^	i.v.	NG	Antiplatelet	8 weeks (100%)	6 months	Fugl-Meyer motor scale, FIM safety outcomes
Moniche 2012 [31]	NRS	Spain	10/10	NIHSS ≥ 8 (15.6), MCA territory	5 to 9 days	Autologous BMMNCs	Mean 3.38 × 10^6^	i.a. ipsilateral MCA	Thrombolysis (30%)	NG	NG	6 months	NIHSS, mRS, BI, safety outcomes

*ALDHbr* aldehyde dehydrogenase, *BI* Barthel index, *BMMNC* bone marrow-derived mononuclear cell, *ESS* European Stroke Scale, *EMS* ESS Motor Subscale, *EPS* endothelial progenitor cell, *EQ-5D* European Quality of Life, *FIM* functional independence measure, *i.a.* intra-arterial infusion, *ICA* internal carotid artery, *i.v.*, intravenous infusion, *MCA* middle cerebral artery, *MRC* Medical Research Council, *MSC* mesenchymal stem cell, *mRS* modified Rankin scale, *NIHSS* National Institute of Health stroke scale, *NG* not given, *PBSC* peripheral blood stem cell, *SC* stem cell, *SSS* Scandinavian Stroke Scale

### Risk of bias in included studies

**Fig. 2:**
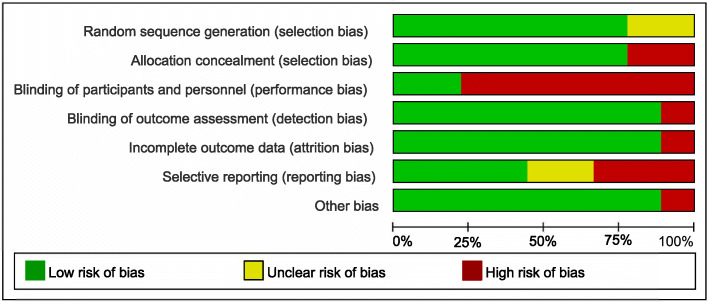
Risk of bias item presented as percentages across all included RCTs

For RCTs [11, 17–24], the Cochrane risk of bias tool was used. Seven RCTs mentioned “random” and described the method of generating a random sequence. Due to the procedures of stem cell transplantation (i.e., bone marrow aspiration or stereotaxic intracerebral implantation), five RCTs were blinded only to outcome assessors and not to participants. Eight RCTs reported that the loss of follow-up was less than 20%. In four RCTs, primary outcomes listed in published protocols were adequately reported in the results. We did not identify any other potential sources of bias in eight RCTs. The detailed assessments are shown in Fig. 2.

For NRSs [25–31], the Newcastle-Ottawa Scale was used. For selection, all the studies were given two stars. For comparability, four studies were given two stars and three studies were given one star. For outcome, six studies were given three stars and one study was given two stars. The overall quality of NRSs was moderate in 5 studies and high in 2 studies (Table 2).

**Table 2:** Assessing the quality of non-randomized studies by Newcastle-Ottawa Scale

	Selection	Comparability	Outcome	Overall quality
Bhasin 2012 [25]	★★	★	★★★	Moderate
Bhasin 2013 [26]	★★	★	★★★	Moderate
Bhasin 2016 [27]	★★	★★	★★	Moderate
Bhasin 2017 [28]	★★	★	★★★	Moderate
Ghali 2016 [29]	★★	★★	★★★	High
Meng 2009 [30]	★★	★	★★★	Moderate
Moniche 2012 [31]	★★	★★	★★★	High

### Efficacy outcomes

#### mRS

**Fig. 3:**
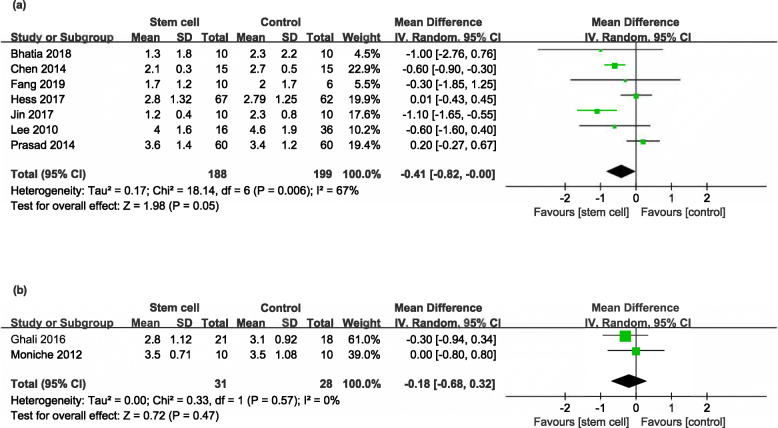
Forest plot of mRS comparing the stem cell group and the control group for RCTs and NRSs. **a** Participants in the stem cell group had a trend beneficial effect in RCTs (MD − 0.41, 95% CI − 0.82 to − 0.00, *I*^*2*^ = 67%). **b** Participants in stem cell group had no beneficial effect in NRSs (MD − 0.81, 95% CI − 0.68 to 0.32, *I*^*2*^ = 0%)

Eight RCTs [17–24] and two NRSs [29, 31] reported the mRS at the end of follow-up (ranged from 6 months to 7 years). But in one RCT [24], the mRS could not be extracted and the corresponding author did not reply to our email inquiries. Participants in the stem cell group had a trend beneficial effect in RCTs (MD − 0.41, 95% CI − 0.82 to − 0.00, *I*^*2*^ = 67%, Fig. 3a), but not in NRSs (MD − 0.81, 95% CI − 0.68 to 0.32, *I*^*2*^ = 0%, Fig. 3b).

#### NIHSS

**Fig. 4:**
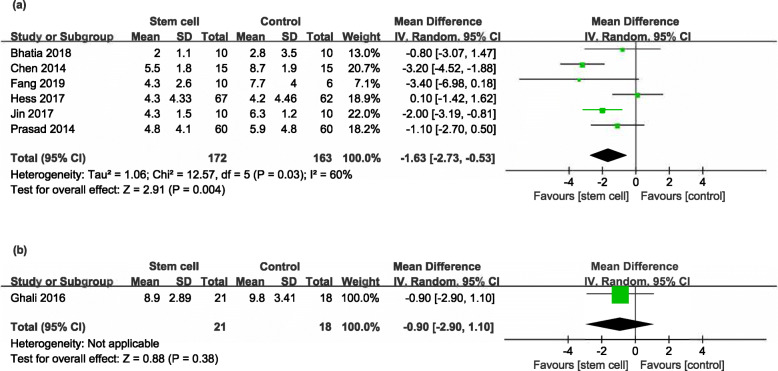
Forest plot of NIHSS comparing the stem cell group and control group for RCTs and NRSs. **a** Participants in the stem cell group had a significantly better outcome compared with controls in RCTs (MD − 1.63, 95% CI − 2.73 to − 0.53, *I*^*2*^ = 60%). **b** Participants in the stem cell group had no beneficial effect in NRSs (MD − 0.90, 95% CI − 2.90 to 1.10, **b**)

Seven RCTs [17–21, 23, 24] and one NRS [29] reported the NIHSS at the end of follow-up (ranged from 6 months to 4 years). But in one RCT [24], the data of NIHSS could not be extracted and the corresponding author did not reply to our email inquiries. Compared with controls, participants in the stem cell group had a significantly better outcome in RCTs (MD − 1.63, 95% CI − 2.73 to − 0.53, *I*^*2*^ = 60%, Fig. 4a), but not in NRS (MD − 0.90, 95% CI − 2.90 to 1.10, Fig. 4b).

#### BI

**Fig. 5:**
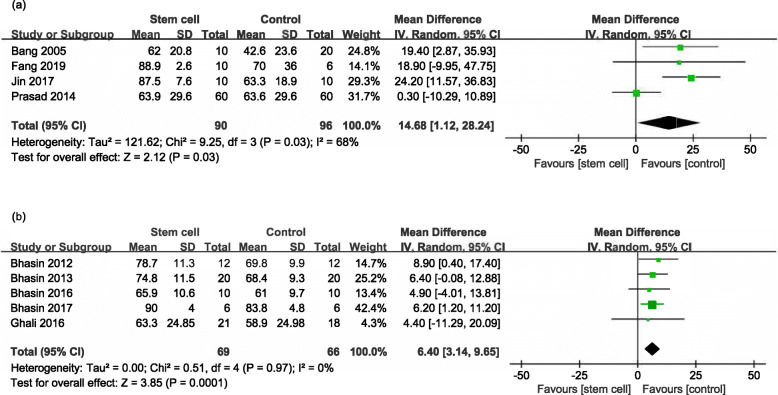
Forest plot of BI comparing the stem cell group and control group for RCTs and NRSs. **a** Stem cell group had a larger effect size than control group in RCTs (MD 14.68, 95% CI 1.12 to 28.24, *I*^*2*^ = 68%). **b** The stem cell group had a larger effect size than the control group in NRSs (MD 6.40, 95% CI 3.14 to 9.65, *I*^*2*^ = 0%)

Five RCTs [11, 19, 21, 23, 24] and five NRSs [25–29] reported the BI at the end of follow-up (ranged from 8 weeks to 7 years). But in one RCT [24], the data of BI could not be extracted and the corresponding author did not reply to our email inquiries. The stem cell group had a larger effect size than the control group in both RCTs (MD 14.68, 95% CI 1.12 to 28.24, *I*^*2*^ = 68%, Fig. 5a) and NRSs (MD 6.40, 95% CI 3.14 to 9.65, *I*^*2*^ = 0%, Fig. 5b).

### Safety outcomes

#### Death

**Fig. 6:**
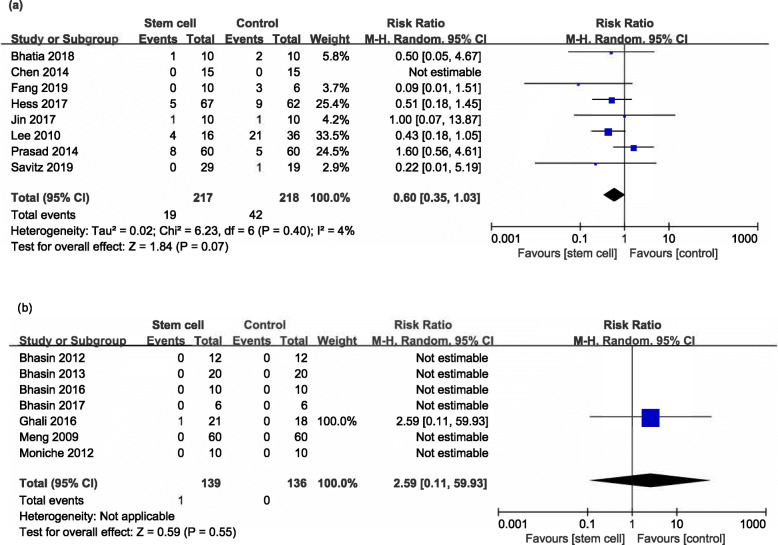
Forest plot of death comparing stem cell group and control group for RCTs and NRSs. **a** There was no significant difference between the stem cell and control group in RCTs (RR 0.60, 95% CI 0.35 to 1.03, *I*^*2*^ = 4%). **b** There was no significant difference between the stem cell and control group in NRSs (RR 2.59, 95% CI 0.11 to 59.93)

Eight RCTs [17–24] and seven NRSs [25–31] reported death at the end of follow-up (ranged from 8 weeks to 7 years). There was no significant difference between the stem cell and control group in RCTs (RR 0.60, 95% CI 0.35 to 1.03, *I*^*2*^ = 4%, Fig. 6a) and NRSs (RR 2.59, 95% CI 0.11 to 59.93, Fig. 6b).

#### Adverse effects

All RCTs and NRSs reported adverse effects at the end of follow-up (ranged from 8 weeks to 7 years). No adverse effect was reported in one RCTs and three NRSs. Fever, headache, and recurrent stroke were the most frequently reported cell-related adverse effects. Other cell-related adverse effects, such as seizures and embolisms, were reported as well. However, neoplasms, tissue overgrowth, and ectopic cell engraftment were not reported. General adverse effects, including psychological illness, renal disorders, and gastrointestinal disorders, were reported. The details of adverse effects reported in each study are shown in Table 3.

**Table 3:** Adverse effects reported in stem cell groups of included studies

Author year	Study type	Cases	Adverse effects (%)
Bang 2005 [11]	RCT	5	Foot cellulitis (20%)
Bhatia 2018 [17]	RCT	10	Death (10%), new infarct (10%)
Chen 2014 [18]	RCT	15	None
Fang 2019 [19]	RCT	10	Deep vein thrombosis (10%), death (10%), atrial fibrillation (20%)
Hess 2017 [20]	RCT	67	Death (7.5%), life-threatening adverse events (4.5%), secondary infections (37.3%), halitosis (9.0%), fever and chills (6.0%), nausea and vomiting (3.0%)
Jin 2017 [21]	RCT	10	Death (10%), fever (20%)
Lee 2010 [22]	RCT	16	Death (25%), seizure (18.8%), ischemic stroke (12.5%), coronary heart disease (6.2%), peripheral artery occlusive disease (6.2%), infection (18.8%), liver enzyme elevation (6.2%), benign tumor (6.2%), neuropyschological illness (37.5%)
Prasad 2014 [23]	RCT	60	Death (13.3%), rise in urea (3.3%), hematological (16.7%), hepatic (36.7%), sensorium deterioration (1.7%), pneumonitis (1.7%), fever (1.7%), hyperglycemia (1.7%), limb ischemia (1.7%), frozen shoulder (3.3%), traumatic injury (1.7%), fracture in lower limb (1.7%), nervous system disorder (10%), gastro intestinal disorder (5%)
Savitz 2019 [24]	RCT	29	Edema peripheral (10.3%), fever (10.3%), chest pain (6.9%), musculoskeletal and connective tissue disorder (37.9%), seizure (13.8%), cerebral hemorrhage (6.9%), cerebral accident (3.5%), dizziness (3.5%), cerebral infarction (3.5%), cerebral ischemia (3.5%), visual field defect (3.5%), psychiatric disorders (44.8%), infections (34.5%), vascular disorders (41.4%), investigations (37.9%), gastrointestinal disorders (24.1%), injury, poisoning, and procedural complications (20.7%), respiratory disorders (20.7%), cardiac disorders (17.2%), erythema (20.7%), nutrition disorders (13.8%), blood disorders (10.3%), renal disorders (10.3%)
Bhasin 2012 [25]	NRS	12	None reported
Bhasin 2013 [26]	NRS	20	None reported
Bhasin 2016 [27]	NRS	10	None reported
Bhasin 2017 [28]	NRS	6	Fever (16.7%), pain (33.3%), seizure (16.7), psychological illness (16.7%)
Ghali 2016 [29]	NRS	21	Death (4.8%), renal impairment (4.8%)
Meng 2009 [30]	NRS	60	Fever (15%), headache (10%)
Moniche 2012 [31]	NRS	10	Seizure (20%), fever (50%), infection (30%), depression (50%), insomnia (30%)

## Discussion

In our review, stem cell-based therapies were related to better outcomes when measured by NIHSS and BI in RCTs and by BI in NRSs. Stem cell group had slightly better in mRS and death but without significant difference. mRS, NIHSS, and BI are widely used scales for stroke in clinical trials. mRS scores range from 0 to 6, which can assess the patient’s functional independence. NIHSS is an 11-item scale which can accurately measure the stroke-related deficits and monitor neurological changes for serial assessment. BI is a 10-item scale to observe patients in a number of key activities of daily living, which can be used to assess the change in patients with stroke [32]. Our result shows that stem cell-based therapies can improve the neurological deficits and activities of daily living in patients with ischemic stroke. But the effect is not enough to produce a significant change on a broader scale.

NIHSS is often used as an inclusion criterion in stroke trials. The inclusion criteria of the studies in our review were too broad (the NIHSS ranged from 4 to 31, Table 1) and the sample size was too small, which diluted the efficacy effect of the stem cell-based therapies. So it is difficult to identify which patients benefit most from the stem cell-based therapies. Most studies included patients with moderate stroke (the mean NIHSS ranged from 9.3 to 15.6, Table 1), but stem cell-based therapies may have uniformly good outcomes for mild stroke. Further studies with narrowing the scope of NIHSS and the suitable population are needed. What is more, patients with the same score on NIHSS may have a different score on mRS or BI. Therefore, the baseline score of mRS or BI may be different. This may have an impact on the evaluation of outcomes based on mRS or BI, especially when the included cases are limited. Only one study evaluated mRS at baseline, and there was a slight improvement on mRS in the stem cell group at the end of follow-up, but the difference was not significant compared with the control group [24]. Further studies need to expand the sample size and evaluate mRS at baseline.

Many factors can influence the effects of stem cell-based therapies in clinical practice. In our review, several cell types, including MSCs, BMMNCs, PBSCs, MultiStem, ALD-401, and EPCs, were used. Moreover, neural stem cells [33], amniotic epithelial cells [34], human adult dental pulp stem cells [35], and umbilical cord blood [36] were also used in primary clinical trials. MSCs are the most extensively investigated cell type because of their potent immunosuppressive effects by producing a multitude of paracrine factors, safety or lack of ethical issues, easy to obtain, lack of immunogenicity, and ability to differentiate into tissue-specific cell line [37]. While majority of the pre-clinical and clinical studies demonstrated significant effects, the clinical significance of these findings was still unclear due to limitations in study design and sample size [38]. Studies using other types of stem cells are limited and mostly in the primary stage; more studies are needed to compare the outcomes in different cell types. Delivery routes in our review were various, including intra-arterial infusion, intravenous infusion, subarachnoid infusion, and stereotaxic implantation. The best route of administration is still unclear. MSC are not free of safety concerns when injected intra-arterially [39]. Intravenous infusion is the minimally invasive method, easy to operate, and has few side effects, so it is widely used in clinical studies [40]. In our review, the number of cells administered in the treatments of stroke were ambiguous, ranging from several million to several billion, and administered once or twice. The smallest dose of stem cells with possible highest benefit and least toxicity is needed [41]. The time window of stem cell transplant was various as well, ranging from 24 h to 2 years covering the acute, subacute, and chronic phase of ischemic stroke. Depending on the cell source/cell type, different time windows of administration may target different mechanisms and lead to a different efficiency [42]. All the factors mentioned above can explain the high heterogeneity in the efficacy outcomes of the included studies.

A recent meta-analysis focused on preclinical studies of MSCs for ischemic stroke showed that MSCs significantly improved all functional outcomes regardless of dose, intravenously administered. MSCs showed significantly greater efficacy in improving motor outcomes. Earlier administration of MSCs before 7 days in rodents might be optimal to enhance functional recovery [43]. Cui and colleagues compared the design differences between preclinical and clinical trials, and recommended freshly harvested, autologous cells and cell transplantation in acute time windows for future clinical studies [44]. These results have important implications for further clinical translation. Further studies must take cell type, cell dose, route of cell administration, and the time window into consideration. And the large scale well-designed clinical trials should follow the guidelines organized by researchers in the academia, industry leaders, and regulatory representatives [45–49]. At the same time, the economic value of stem cell-based therapies in the treatment of ischemic stroke should be evaluated [50]. In addition, systematic analyses of clinical trial results usually focus on the functional recovery rather than infarct volume in preclinical studies. Further preclinical studies should select appropriate functional tests for the respective stroke model, species, scenario, and study duration [49].

Previous studies summarized adverse events that had been discovered in preclinical and clinical investigations assessing cell therapies for stroke [51]. Fever, headache, and stroke recurrence were frequently reported cell-related adverse effects in our review. But there was no significant difference compared with the control group according to previous results [13, 14]. Antithrombotic treatment can reduce the risk of recurrent stroke, but only four RCTs and one NRSs reported antithrombotic treatment. The irregular use of antithrombotic treatment may be the cause of the high recurrent of stroke. For patients with symptomatic intracranial atherosclerotic disease, aggressive medical therapy is needed [52]. Rehabilitation can reduce the risk of adverse effects such as medical morbidity and psychological illness [53]. But only three RCTs and five NRSs in our review reported rehabilitation therapies. What is more, mind-body movements such as yoga and tai chi are useful alternative rehabilitation measures [54, 55]. Further stem cell studies need to take standardized medical and rehabilitation therapies into account, because those may reduce the risk of adverse effects.

Extensive attempts have been made to improve the efficacy of stem cell-based therapies for ischemic stroke and reduce the risk of adverse effects recently. Strategies to enhance the endurance and efficacy of grafted stem cells in ischemic conditions include treating with growth factors, pharmacological agents, ischemia/hypoxia, or electrical stimulation and have increased paracrine potentials [56–58]. Stem cells modified with exogenous growth factor genes such as vascular endothelial growth factor; brain-derived neurotrophic factor by viral or non-viral delivery system can significantly increase the paracrine effects and decrease the mortality of mice [59, 60]. Implantation of modified bone marrow-derived mesenchymal stem cells (SB623) transiently transfected with the human Notch-1 intracellular domain in a patient with stable chronic stroke is safe and is accompanied by improvements in clinical outcomes [61]. It is now well established that there is no engraftment of MSCs in the brain after intravenous administration and the recovery effects observed in some ischemic animal models are mediated by factors secreted by MSCs [62]. Recently, extracellular vesicles released by MSCs or NSCs are recognized as effective in vivo. Compared to stem cells, they have similar effects but with lower risk (in terms of vessel occlusion) [63, 64]. Extracellular vesicles are emerging as a novel alternative to cell therapy [65].

Our review has several limitations. Clinical trials of stem cell-based therapies for ischemic stroke are still in early stage. The number of cases in the stem cell group was less than thirty in most included studies. There was high heterogeneity in the efficacy outcomes of RCTs. Many factors such as cell types, cell numbers, delivery routes, time window, and medical and rehabilitation therapies affect the efficacy of stem cells. The explorations of the sensitivity and heterogeneity were not feasible owing to the small number of included studies. We failed to extract the efficacy outcomes of a recent RCT because the outcome indicators were unclear and the author was not contacted.

## Conclusion

In our review, stem cell-based therapies can improve the neurological deficits and activities of daily living in patients with ischemic stroke, but the benefits are still limited. At present, the clinical trial of stem cell-based therapies for ischemic stroke is still in the early stage, and participants are still limited. Further clinical trials are needed to find out the suitable population and explore the best option of stem cell-based therapy for ischemic stroke.

## Supplementary information

**Additional file 1.** PRISMA 2009 checklist.

**Additional file 2.** Search strategy for PubMed.

**Additional file 3 Table A1.** Characteristics of studies excluded from further analysis.

## Data Availability

Not applicable.

## Abbreviations

ALDHbr: Aldehyde dehydrogenase
BI: Barthel index
BMMNC: Bone marrow-derived mononuclear cell
ESS: European Stroke Scale
EMS: ESS Motor Subscale
EPS: Endothelial progenitor cell
EQ-5D: European Quality of Life
FIM: Functional independence measure
i.a.: Intra-arterial infusion
ICA: Internal carotid artery
i.v.: Intravenous infusion
MCA: Middle cerebral artery
MRC: Medical Research Council
MSC: Mesenchymal stem cell
mRS: Modified Rankin scale
NIHSS: National Institute of Health stroke scale
NG: Not given
PBSC: Peripheral blood stem cell
SC: Stem cell
SSS: Scandinavian Stroke Scale
CI: Confidence interval
MD: Mean difference
NRS: Non-randomized study
RCT: Randomized controlled trial
RR: Risk ratio
