# Supplementary material for: Stem cell-based therapies for ischemic stroke: a systematic review and meta-analysis of clinical trials
Source: Stem Cell Res Ther. 2020 Jun 26;11:252. doi: 10.1186/s13287-020-01762-z (PMC7318436; doi:10.1186/s13287-020-01762-z)
Supplement: Supplementary file 2 — Additional file 2. Search strategy for PubMed. [file 13287_2020_1762_MOESM2_ESM.docx]

**Search strategy for PubMed**

1 “clinical trial”[Publication Type] OR "placebo"[tiab] OR "intervention study"[tiab] OR "trial"[tiab] OR "groups"[tiab]

2 “stroke”[MeSH] OR "apoplexy"[tiab] OR "cerebrovascular accident*"[tiab] OR "brain vascular accident*"[tiab] OR "stroke*"[tiab] OR "cerebral infarct*"[tiab] OR "brain ischemia"[MeSH] OR "brain ischemia*"[tiab] OR "cerebral ischemia*"[tiab] OR "ischemic encephalopathy"[tiab] OR "brain infarct*"[tiab]

3 "cell- and tissue-based therapy"[MeSH] OR "stem cells"[MeSH] OR "cell therapy"[tiab] OR "cellular therapy"[tiab] OR "cell transplantation"[tiab] OR "cellular transplantation"[tiab] OR "cell delivery"[tiab] OR "cellular delivery"[tiab] OR "cell infusion"[tiab] OR "cellular infusion"[tiab] OR "stem cell"[tiab] OR "stem cells"[tiab]

1 AND 2 AND 3
