# Supplementary material for: Stem cell-based therapies for ischemic stroke: a systematic review and meta-analysis of clinical trials
Source: Stem Cell Res Ther. 2020 Jun 26;11:252. doi: 10.1186/s13287-020-01762-z (PMC7318436; doi:10.1186/s13287-020-01762-z)
Supplement: Supplementary file 3 — Additional file 3 Table A1. Characteristics of studies excluded from further analysis. [file 13287_2020_1762_MOESM3_ESM.docx]

Table A1. Characteristics of studies excluded from further analysis

| Author year | Reason for exclusion |
| --- | --- |
| Suarez-Monteagudo 2009 (1) | single-arm study |
| Battistella 2011 (2) | single-arm study |
| Bentley 2011 (3) | single-arm study |
| Chung 2011 (4) | single-arm study |
| Honmou 2011 (5) | single-arm study |
| Padma 2011 (6) | single-arm study |
| Chen 2012 (7) | single-arm study |
| Friedrich 2012 (8) | single-arm study |
| Vahidy 2012 (9) | single-arm study |
| Chen 2013 (10) | single-arm study |
| Jiang 2013 (11) | single-arm study |
| Lu 2013 (12) | single-arm study |
| Banerjee 2014 (13) | single-arm study |
| Qiao 2014 (14) | single-arm study |
| Sharma 2014 (15) | single-arm study |
| Taguchi 2015 (16) | single-arm study |
| Honmou 2016 (17) | single-arm study |
| Kalladka 2016 (18) | single-arm study |
| Kurtzberg 2016 (19) | single-arm study |
| Lin 2016 (20) | single-arm study |
| Bulters 2017 (21) | single-arm study |
| Laskowitz 2018 (22) | single-arm study |
| Steinberg 2018 (23) | single-arm study |
| Haque 2019 (24) | single-arm study |
| Mohamed Ibrahim 2016 (25) | ongoing study |
| Nagpal 2016 (26) | ongoing study |
| Khurana 2017 (27) | ongoing study |
| Moniche 2017 (28) | ongoing study |
| Shichinohe 2017 (29) | ongoing study |
| Kim 2018 (30) | ongoing study |
| Osanai 2018 (31) | ongoing study |
| Phan 2018 (32) | ongoing study |
| Sargento-Freitas 2018 (33) | ongoing study |
| Deng 2019 (34) | ongoing study |
| NCT00875654 | ongoing study |
| NCT01091701 | ongoing study |
| EUCTR2011-001393-26-ES | ongoing study |
| NCT01518231 | ongoing study |
| JPRN-JMA-IIA00117 | ongoing study |
| CHICTR-TRC-14005071 | ongoing study |
| NCT01678534 | ongoing study |
| EUCTR2013-002135-15-ES | ongoing study |
| IRCT2014070817867N2 | ongoing study |
| NCT02425670 | ongoing study |
| NCT02580019 | ongoing study |
| NCT02448641 | ongoing study |
| NCT02378974 | ongoing study |
| NCT02813512 | ongoing study |
| JPRN-UMIN000026130 | ongoing study |
| NCT03186456 | ongoing study |
| NCT03384433 | ongoing study |
| NCT03176498 | ongoing study |
| NCT03629275 | ongoing study |
| ACTRN12618001094268 | ongoing study |
| ISRCTN15677760 | ongoing study |

References

1. Suarez-Monteagudo C, Hernandez-Ramirez P, Alvarez-Gonzalez L, Garcia-Maeso I, de la Cuetara-Bernal K, Castillo-Diaz L, et al. Autologous bone marrow stem cell neurotransplantation in stroke patients. An open study. Restor Neurol Neurosci. 2009;27(3):151-61.

2. Battistella V, de Freitas GR, da Fonseca LM, Mercante D, Gutfilen B, Goldenberg RC, et al. Safety of autologous bone marrow mononuclear cell transplantation in patients with nonacute ischemic stroke. Regen Med. 2011;6(1):45-52.

3. Bentley P, Banerjee S, Hamady M, Gordon M, Mehta A, Waldman A, et al. UK Stroke Forum 2011 Abstracts, Glasgow, 29 November 2011–1 December 2011. International Journal of Stroke. 2011;6(2_suppl):1-65.

4. Chung D, Lee YB, Kim YS, Kim HY, Koh SH. 20th European Stroke Conference, Hamburg, Germany, May 24-27, 2011. Cerebrovasc Dis. 2011;31 Suppl 2:1-341.

5. Honmou O, Houkin K, Matsunaga T, Niitsu Y, Ishiai S, Onodera R, et al. Intravenous administration of auto serum-expanded autologous mesenchymal stem cells in stroke. Brain. 2011;134(Pt 6):1790-807.

6. Padma MV, Bhasin A, Bhatia R, Kumaran S, Mohanty S. 20th European Stroke Conference, Hamburg, Germany, May 24-27, 2011. Cerebrovasc Dis. 2011;31 Suppl 2:1-341.

7. Chen WM, Zou QY, Lu JJ, Hu YX, Hu QL, Li ZG, et al. Reinfusion of autologous bone marrow mesenchymal stem cells for treatment of stroke in 30 cases. Chinese Journal of Tissue Engineering Research. 2012;16(32):6071-5.

8. Friedrich MA, Martins MP, Araujo MD, Klamt C, Vedolin L, Garicochea B, et al. Intra-arterial infusion of autologous bone marrow mononuclear cells in patients with moderate to severe middle cerebral artery acute ischemic stroke. Cell Transplant. 2012;21 Suppl 1:S13-21.

9. Vahidy F, Kar S, Aisiku I, Juneja H, Lee D, Garret J, et al. Abstracts for the 19th Annual Meeting of the American Society for Neural Therapy and Repair. Cell Transplant. 2012;21(4_suppl):773-97.

10. Chen L, Xi H, Huang H, Zhang F, Liu Y, Chen D, et al. Multiple cell transplantation based on an intraparenchymal approach for patients with chronic phase stroke. Cell Transplant. 2013;22 Suppl 1:S83-91.

11. Jiang Y, Zhu W, Zhu J, Wu L, Xu G, Liu X. Feasibility of delivering mesenchymal stem cells via catheter to the proximal end of the lesion artery in patients with stroke in the territory of the middle cerebral artery. Cell Transplant. 2013;22(12):2291-8.

12. Lu WS, Li ZC, Tian ZM, Jia B, Zeng YJ. Clinical Transplantation of Human Embryonic Neural Stem Cells for the Treatment of Cerebral Infarction Sequelae. Neurosurgery Quarterly. 2013;23(1):58-60.

13. Banerjee S, Bentley P, Hamady M, Marley S, Davis J, Shlebak A, et al. Intra-Arterial Immunoselected CD34+ Stem Cells for Acute Ischemic Stroke. Stem Cells Transl Med. 2014;3(11):1322-30.

14. Qiao LY, Huang FJ, Zhao M, Xie JH, Shi J, Wang J, et al. A two-year follow-up study of cotransplantation with neural stem/progenitor cells and mesenchymal stromal cells in ischemic stroke patients. Cell Transplant. 2014;23 Suppl 1:S65-72.

15. Sharma A, Sane H, Gokulchandran N, Khopkar D, Paranjape A, Sundaram J, et al. Autologous bone marrow mononuclear cells intrathecal transplantation in chronic stroke. Stroke Res Treat. 2014;2014:234095.

16. Taguchi A, Sakai C, Soma T, Kasahara Y, Stern DM, Kajimoto K, et al. Intravenous Autologous Bone Marrow Mononuclear Cell Transplantation for Stroke: Phase1/2a Clinical Trial in a Homogeneous Group of Stroke Patients. Stem Cells Dev. 2015;24(19):2207-18.

17. Honmou O. Phase III clinical trial using autologous mesenchymal stem cells for stroke patients. Nihon rinsho [japanese journal of clinical medicine]. 2016;74(4):649‐54.

18. Kalladka D, Sinden J, Pollock K, Haig C, McLean J, Smith W, et al. Human neural stem cells in patients with chronic ischaemic stroke (PISCES): a phase 1, first-in-man study. Lancet. 2016;388(10046):787-96.

19. Kurtzberg J, Troy JD, Bennett E, Belagaje S, Shpall EJ, Wiese J, et al. Allogeneic Umbilical Cord Blood Infusion for Adults with Ischemic Stroke (CoBIS): Clinical Outcomes from a Phase 1 Safety Study. Blood. 2016;128(22).

20. Lin CH, Chui L, Tsai CH, Chen JC, Cho DY, Hsu CY, et al. Asia Pacific Stroke Conference 2016. Abstracts of the Annual Conference of theAsia Pacific Stroke Organization (APSO) Combined with Stroke Society of Australasia, Brisbane, Qld., Australia, July 14-17, 2016: Abstracts. Cerebrovasc Dis. 2016;42 Suppl 1:1-157.

21. Bulters D, Wilmot M, Sprigg N, Dixit A, Ward N, Tyrrell P, et al. Late Breaking Abstracts. European Stroke Journal. 2017;2(1_suppl):477-95.

22. Laskowitz DT, Bennett ER, Durham RJ, Volpi JJ, Wiese JR, Frankel M, et al. Allogeneic Umbilical Cord Blood Infusion for Adults with Ischemic Stroke: Clinical Outcomes from a Phase I Safety Study. Stem Cells Transl Med. 2018;7(7):521-9.

23. Steinberg GK, Kondziolka D, Wechsler LR, Lunsford LD, Kim AS, Johnson JN, et al. Two-year safety and clinical outcomes in chronic ischemic stroke patients after implantation of modified bone marrow-derived mesenchymal stem cells (SB623): a phase 1/2a study. J Neurosurg. 2018:1-11.

24. Haque ME, Gabr RE, George SD, Boren SB, Vahidy FS, Zhang X, et al. Serial Cerebral Metabolic Changes in Patients With Ischemic Stroke Treated With Autologous Bone Marrow Derived Mononuclear Cells. Front Neurol. 2019;10:141.

25. Mohamed Ibrahim N, Tan H, Chin S, Law Z, Ismail N, Amran@Azman N, et al. BM-MSC Accelerates Acute Stroke Recovery in a Randomized Placebo-Controlled Clinical Phase II/III Study. Cytotherapy. 2016;18(6):S8.

26. Nagpal A, Kremer KL, Hamilton-Bruce MA, Kaidonis X, Milton AG, Levi C, et al. TOOTH (The Open study Of dental pulp stem cell Therapy in Humans): Study protocol for evaluating safety and feasibility of autologous human adult dental pulp stem cell therapy in patients with chronic disability after stroke. Int J Stroke. 2016;11(5):575-85.

27. Khurana D, Singh R, Gupta V, Sharma R, Khandelwal N. Late Breaking Abstracts. European Stroke Journal. 2017;2(1_suppl):477-95.

28. Moniche F, Escudero I, Zapata-Arriaza E, Calderon-Cabrera C, Martin-Sanchez J, Vega-Salvatierra A, et al. Poster Abstracts. European Stroke Journal. 2017;2(1_suppl):98-476.

29. Shichinohe H, Kawabori M, Iijima H, Teramoto T, Abumiya T, Nakayama N, et al. Research on advanced intervention using novel bone marrOW stem cell (RAINBOW): a study protocol for a phase I, open-label, uncontrolled, dose-response trial of autologous bone marrow stromal cell transplantation in patients with acute ischemic stroke. BMC Neurol. 2017;17(1):179.

30. Kim OJ. A Randomized, Double-blind, Placebo-controlled, Phase I / IIa Clinical Trial for Evaluation of the Safety and Potential Therapeutic Effects After Intravenous Transplantation of Human Umbilical Cord-derived Mesenchymal Stem Cells in Patients With Cerebral Infarction. Stroke. 2018;49.

31. Osanai T, Houkin K, Uchiyama S, Minematsu K, Taguchi A, Terasaka S. Treatment evaluation of acute stroke for using in regenerative cell elements (TREASURE) trial: Rationale and design. Int J Stroke. 2018;13(4):444-8.

32. Phan TG, Ma H, Lim R, Sobey CG, Wallace EM. Phase 1 Trial of Amnion Cell Therapy for Ischemic Stroke. Front Neurol. 2018;9(JUN):198.

33. Sargento-Freitas J, Pereira A, Gomes A, Amorim P, Matos T, Cardoso CMP, et al. STROKE34 Study Protocol: A Randomized Controlled Phase IIa Trial of Intra-Arterial CD34+ Cells in Acute Ischemic Stroke. Front Neurol. 2018;9:302.

34. Deng L, Peng Q, Wang H, Pan J, Zhou Y, Pan K, et al. Intrathecal Injection of Allogenic Bone Marrow-Derived Mesenchymal Stromal Cells in Treatment of Patients with Severe Ischemic Stroke: Study Protocol for a Randomized Controlled Observer-Blinded Trial. Transl Stroke Res. 2019;10(2):170-7.
